# Identification of novel targets for host-directed therapeutics against intracellular *Staphylococcus aureus*

**DOI:** 10.1038/s41598-019-51894-3

**Published:** 2019-10-28

**Authors:** Natalia Bravo-Santano, Pablo Capilla-Lasheras, Luis M. Mateos, Yolanda Calle, Volker Behrends, Michal Letek

**Affiliations:** 1 Health Sciences Research Centre, University of Roehampton, London, UK; 2 Centre for Ecology and Conservation, University of Exeter, Penryn Campus, Cornwall, UK; 3 Department of Molecular Biology, Area of Microbiology, University of León, León, Spain

**Keywords:** Bacterial host response, Cellular microbiology

## Abstract

During patient colonization, *Staphylococcus aureus* is able to invade and proliferate within human cells to evade the immune system and last resort drugs such as vancomycin. Hijacking specific host molecular factors and/or pathways is necessary for pathogens to successfully establish an intracellular infection. In this study, we employed an unbiased shRNA screening coupled with ultra-fast sequencing to screen 16,000 human genes during *S. aureus* infection and we identified several host genes important for this intracellular pathogen. In addition, we interrogated our screening results to find novel host-targeted therapeutics against intracellular *S. aureus*. We found that silencing the human gene *TRAM2* resulted in a significant reduction of intracellular bacterial load while host cell viability was restored, showing its importance during intracellular infection. Furthermore, TRAM2 is an interactive partner of the endoplasmic reticulum SERCA pumps and treatment with the SERCA-inhibitor Thapsigargin halted intracellular MRSA survival. Our results suggest that Thapsigargin could be repurposed to tackle *S. aureus* host cell infection in combination with conventional antibiotics.

## Introduction

*Staphylococcus aureus* is a facultative intracellular pathogen capable of surviving in a wide range of human cells^1^. It is becoming clear that the intracellular survival of *S. aureus* has an important role on nasal colonization^2^. In persistent carriers, this could lead to opportunistic infections, increasing the risk of mortality and medical costs^3^. Moreover, intracellular *S. aureus* may contribute to persistent rhinitis, recurrent tonsillitis and chronic osteomyelitis^4–8^. Importantly, many last resort antibiotics do not enter efficiently into the host cell to achieve intracellular killing^9^. Therefore, novel strategies to control intracellular *S. aureus* and patient decolonization are urgently needed^2^.

There are different strategies available to determine host factors hijacked by intracellular pathogens, combining genomics, computational biology, proteomics and transcriptomics^10^. These strategies could lead to the identification or development of new host-targeted treatments to combat intracellular infections. In particular, loss-of-function phenotypic analysis, such as RNA interference (RNAi), is a common procedure to identify host genes or proteins that are necessary for the pathogen internalization, growth or survival within mammalian cells^11–13^.

The assessment of gene function using RNAi approaches relies on degradation of specific messenger RNAs (mRNAs) to silence gene expression. In mammalian cells, RNAi is usually mediated by microRNAs (miRNAs) that are non-protein coding transcripts. shRNAs are miRNAs that are processed into shorter RNAs and contain a short-hairpin structure. These shRNAs are then further processed into short double-stranded pieces of RNA called short-interfering RNAs (siRNAs). The molecular mechanism of gene silencing comprises the binding of one strand of the siRNA duplex to a protein-coding mRNA transcript within the multi-protein RNA-Induced Silencing Complex (RISC). Consequently, this interaction triggers cleavage of the protein-coding mRNA by a nuclease in the RISC complex, thereby destroying the mRNA and silencing the expression of the gene^14^.

One of the main advantages of using lentiviral vectors encoding and expressing shRNAs, when compared to siRNAs, is their ability to stably integrate into the genome of mammalian cells, allowing the generation of established knockdown cell lines (e.g. after puromycin selection) and therefore longer experimental settings^14,15^. Furthermore, the availability of commercial shRNA libraries constitutes a powerful tool that allows large-scale loss-of-function genetic screenings in mammalian cells^14,16^.

We recently identified new host-directed therapeutics against intracellular *S. aureus* by means of metabolomics and high-throughput drug screening^17,18^. In this study, we employed an shRNA screening approach coupled to Illumina sequencing-based deconvolution to identify novel host-genes that could be involved in *S. aureus* cell infection. Our results indicate that TRAM2 is a promising host target for the development of new anti-infectives against intracellular MRSA.

## Results

### Identification of novel host-genes involved in *S. aureus* cell infection

**Figure 1:**
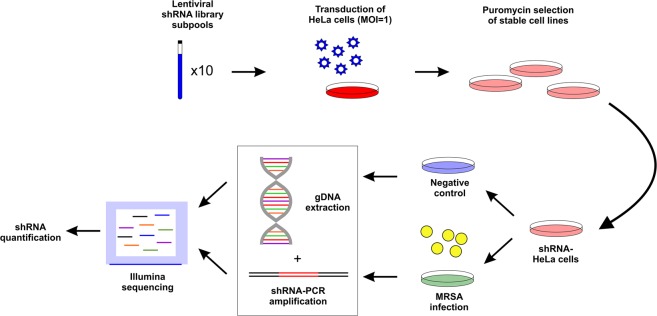
Layout of shRNA screening approach. HeLa cells were first transduced with shRNA library until stable cells were produced (shRNA-HeLa). shRNA-HeLa cells were infected with MRSA and genomic DNA was extracted to determine by Illumina sequencing the occurrence of each shRNA after infection. Uninfected HeLa cells were used as negative control.

We tested the effect of silencing 16,000 human genes during *S. aureus* cell infection by using the Mission® LentiPlex^®^ Human Pooled shRNA Library (Fig. 1). Most of the genes were targeted by at least 5 different shRNA constructs to ensure reproducibility (Supplementary Fig. 1). We transduced HeLa cells with the shRNA library and performed puromycin selection to obtain cells stably expressing the library (shRNA-HeLa cells; Fig. 1). Subsequently, we infected shRNA-HeLa cells with the clinically relevant *S. aureus* NCTC 13626 strain to investigate the effect of silencing specific genes on intracellular MRSA infection; vancomycin was added after one hour of incubation to kill extracellular bacteria^17^, and uninfected shRNA-HeLa cells were used as negative control. After six hours of infection, we extracted total genomic DNA to determine by Illumina sequencing the total occurrence of each shRNA integrated on the shRNA-HeLa cells after infection (Fig. 1). By calculating the ratio between the occurrence of all shRNA constructs targeting the same gene found in *S. aureus*-infected cells (sample) versus the uninfected condition (control) (Supplementary Table 1), we identified 5,674 shRNAs that were under-represented after *S. aureus* infection, whereas 9,997 shRNAs were over-represented when compared to uninfected cells (Supplementary Fig. 2).

Host cellular death is the last step of a successful intracellular infection, thus over-represented shRNA vectors may select for host cells that halt intracellular MRSA by silencing host genes that support the cell invasion or intracellular replication of *S. aureus*. In other words, over-represented shRNA vectors indicate an increase in host cell viability (via their copy number in the population of infected cells). The targets of these over-represented shRNAs, i.e. transcripts encoding proteins used by the pathogen to enter or proliferate inside the host cells, represent the main focus of our downstream analysis.

**Figure 2:**
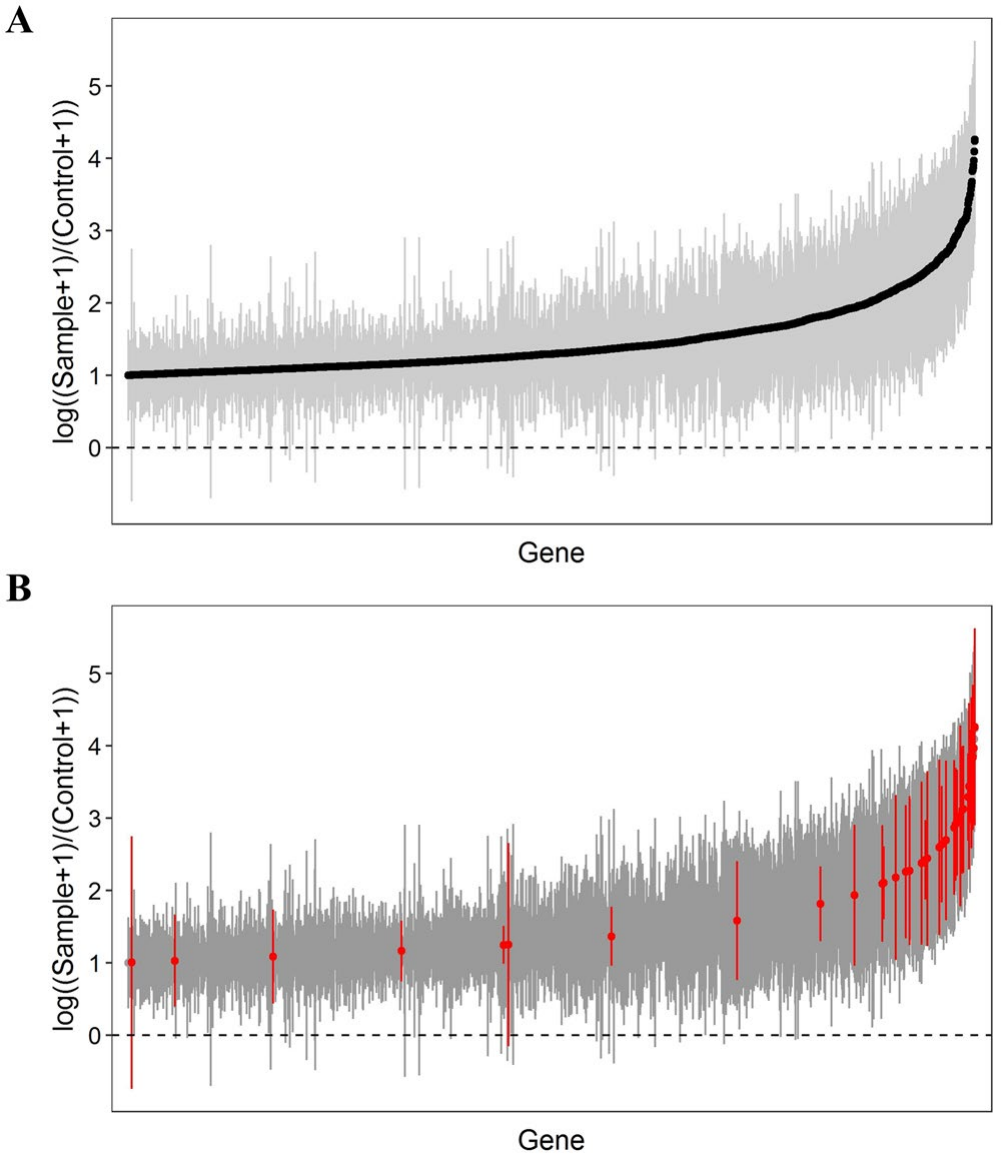
shRNA screening data after applying a strict cut-off. (**A**) shRNA screening data were filtered by establishing three criteria: mean ratio higher than 1 (Log Ratio >1) compared to the uninfected control, standard error lower than 2 (SE <2) and a consistency higher than 60%. (**B**) Out of 2,888 genes, twenty-nine host genes were selected for further validation. High effect in the shRNA screening after MRSA infection and/or a biological relevance for intracellular pathogenesis were used as criteria to choose the most interesting hits. Selected genes are highlighted in red.

As an initial step, shRNA abundance/host cell viability data were analysed based on their significance and magnitude of change, and 38 transcript targets were highlighted after applying a strict cut-off (Supplementary Fig. 3). However, the silencing effect of empirically-designed shRNA vectors could be limited by their variable efficiency and specificity^19^. Therefore, we further interrogated our screening results by considering the percentage of shRNAs that were under- or over-represented for each gene to estimate the consistency of the silencing effect. This is important because a consistent silencing effect produced by multiple shRNA constructs targeting the same gene is less likely to be due to an off-target effect^15^. We then established several cut-offs to identify the most interesting gene candidates in our model of infection, which included a mean ratio greater than 1 (Log Ratio >1) compared to uninfected cells, a standard error below 2 (SE <2) and a consistency over 60% (i.e. 60% of gene-specific shRNAs caused an effect in the same direction). In total, 2,888 (≈18% of all genes) met these criteria (Fig. 2A). The identified genes were functionally classified into 63 cellular pathways using DAVID Bioinformatics Resources 6.8^20^ (Supplementary Fig. 4 and Supplementary Table 2). We identified overrepresented pathways that may be important during *S. aureus* cell infection, e.g. regulation of autophagy, regulation of actin cytoskeleton, MAPK signalling and Toll-like receptor signalling pathways.

Following the aforementioned principles (i.e. magnitude and significance of change, consistent silencing effect and biological relevance), we selected a total of 29 genes for further validation (Fig. 2B and Supplementary Table 3). By choosing these genes, we covered a diverse range of cellular pathways known to be exploited by MRSA during intracellular infection and - importantly - novel genes whose functions (and/or involvement in MRSA invasion) may not have been thoroughly described.

Among the shortlisted genes candidates, the Autophagy Related 10 (*ATG10*) gene is directly involved in cell autophagy^21,22^. Other genes are associated with programmed cell death, such as Apoptosis and Caspase Activation Inhibitor (*AVEN*) and CD5 molecule-like (*CD5L*) genes. *RAB* genes are linked to membrane trafficking^23^ and, indeed, several intracellular pathogens hijack host-RAB proteins during infection^24^. The components of the cell cytoskeleton also play an important role during intracellular infection, and we identified at least three proteins, CLTB and MYL2/9, which may be involved in cytoskeleton rearrangement^25^. We also observed proteins linked to the host cell metabolism, such as the branched-chain amino acids regulator (BCKDK) and the amino acids transporters SLC43A1 and SLC63A3, which could be important for intracellular MRSA survival^17^. Additionally, we also included the CD83-molecule (*CD83*) and Toll-Like Receptor 2 (*TLR2*) genes in our downstream analysis, which are involved in a wide range of host immune responses to fight against bacterial and viral infections^26,27^.

To validate our results, we used individual shRNA constructs with the strongest silencing effect for each gene to produce individual knockdowns in HeLa cells for further investigation. An established cell line expressing an shRNA that does not silence any human gene (Non-target HeLa cells) was used as a negative control in all downstream experiments.

**Figure 3:**
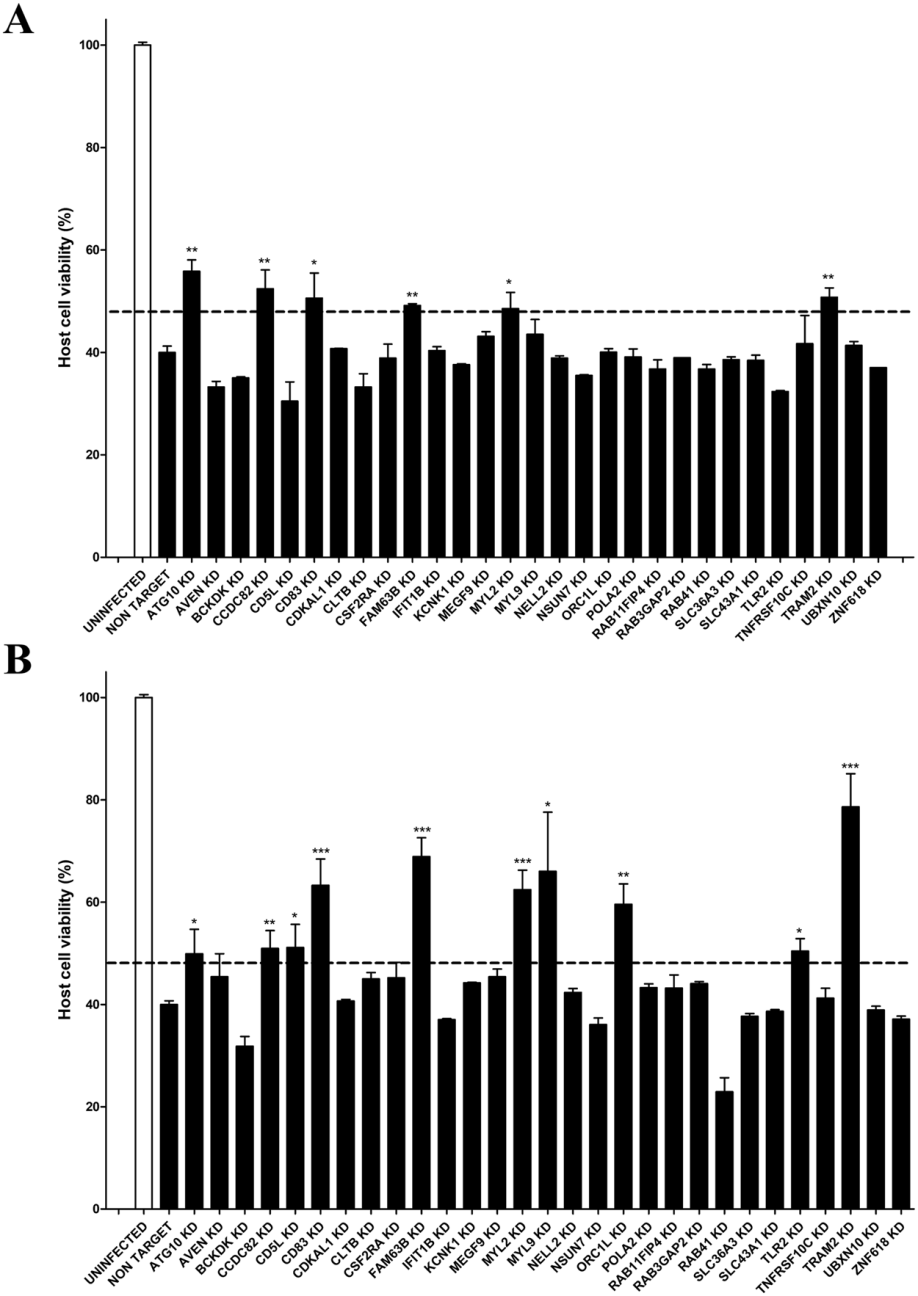
Validation of the screening results. Host cell viability after intracellular infection with *S. aureus* NCTC 13626 strain (**A**) and *S. aureus* USA300 LAC strain (**B**). After 6 hours of MRSA infection, cell viability was quantified by flow cytometry using a double staining of Annexin V-FITC and propidium iodide. Cell viability was normalized in relation to the uninfected control. Data are expressed by means ± standard error (SE) of two biological replicates performed in duplicates. One-way ANOVA and multiple comparison Tukey’s tests were performed to assess statistically significant differences between each knockdown and the non-target control. p-value ≤ 0.05 (*); ≤0.01 (**); ≤0.001 (***). In order to recognize the most interesting hits, we selected knockdowns whose cell viability was at least 1.2-fold higher than non-target cells after infection with both strains.

We first assessed the host cell viability of the selected 29 knockdowns after MRSA infection (Fig. 3 and Supplementary Table 3). We performed infection assays with two different strains of MRSA – *S. aureus* NCTC 13626 (Healthcare-Acquired MRSA) and *S. aureus* USA300 LAC (Community-Acquired MRSA; Fig. 3) – in the presence of vancomycin and gentamycin respectively, since NCTC 13626 is resistant to gentamycin^17^. We selected *CD83, FAM63B, MYL2* and *TRAM2* knockdowns for further analysis since the silencing of these four host genes resulted in increased host cell viability regardless of the strain that was employed, and these genes were not studied before in relation to *S. aureus* cell infection.

**Figure 4:**
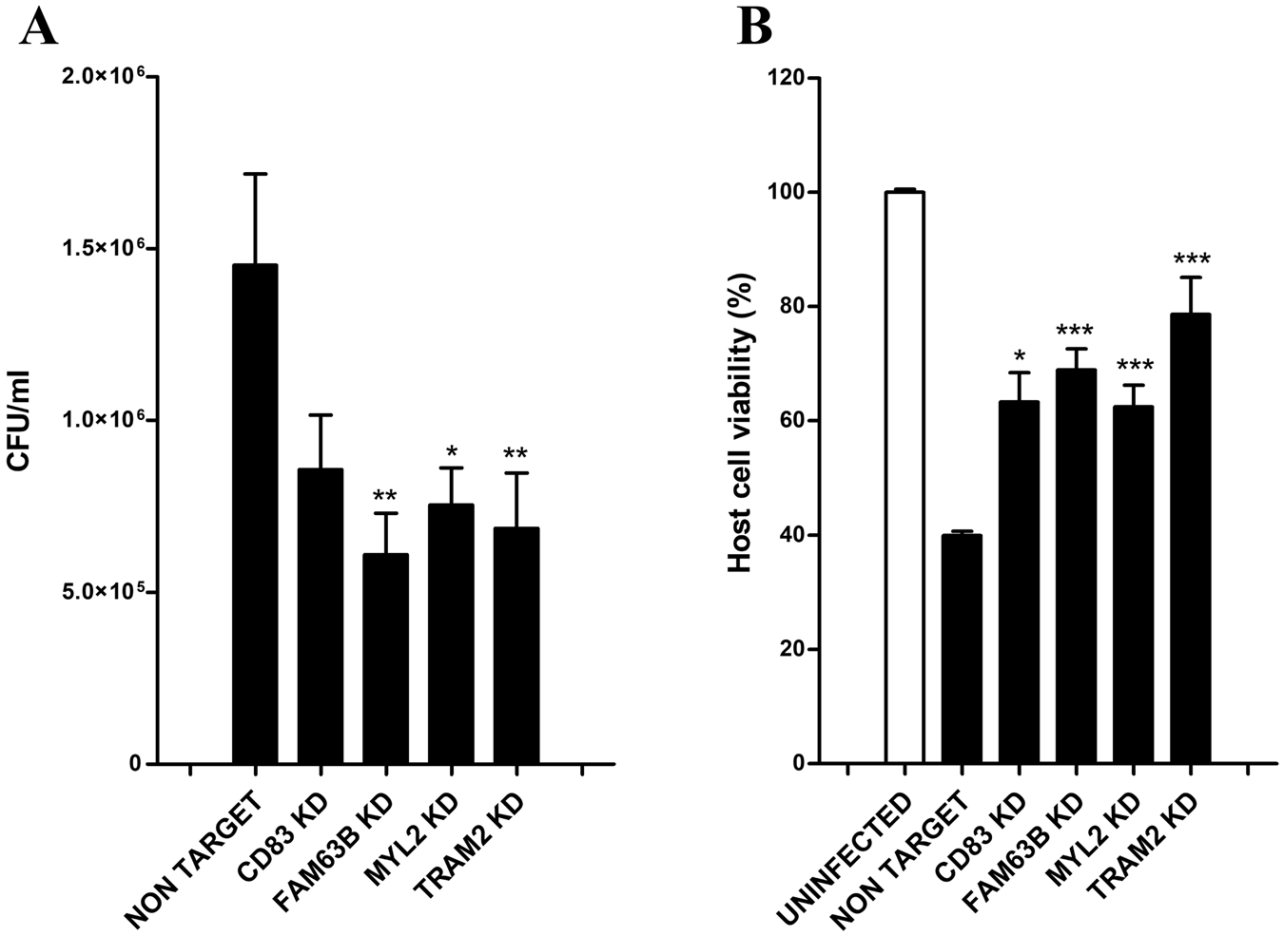
Bacterial and host cell viability after *S. aureus* USA300 infection in *CD83, FAM63B, MYL2*, and *TRAM2* knockdowns. (**A**) Intracellular bacteria were quantified by colony forming units (CFU) after 6 hours of infection. (**B**) Cell viability was quantified in parallel after 6 hours of infection by flow cytometry, using a double staining of Annexin V-FITC and propidium iodide. Uninfected HeLa cells were employed as negative control and cell viability was normalized in relation to the control’s viability. Data are expressed by means ± standard error (SE) of three experiments performed in duplicates. One-way ANOVA and multiple comparison Tukey’s tests were performed to assess statistically significant differences between each knockdown and the non-target control. p-value ≤ 0.05 (*); ≤0.01 (**); ≤0.001 (***).

We then assessed the intracellular bacterial load of *S. aureus* USA300 in *CD83, FAM63B, MYL2* and *TRAM2* knockdowns in parallel to host cell viability (Fig. 4). We observed a clear inhibitory effect on the intracellular growth in every knockdown tested in comparison to the non-targeted control, with the most pronounced effects after silencing *FAM63B, MYL2* or *TRAM2* (Fig. 4A). We finally found drug inhibitors linked to two proteins that were consistently identified in all tests: MYL2 and TRAM2, which were chosen for further analysis.

### Thapsigargin impairs intracellular *S. aureus* infection

Blebbistatin inhibits the ATPase activity of myosin II and stops motility based on actomyosin association^28^. However, the treatment with this inhibitor had no effect on the host cell viability during infection (Supplementary Fig. 5), discarding *MYL2* for further downstream experiments.

On the other hand, TRAM2 is part of the translocon, a complex of proteins involved in the transport of polypeptides across the endoplasmic reticulum (ER) membrane^29^. The C-terminal end of TRAM2 interacts with the Ca^2+^ pump of the endoplasmic reticulum SERCA2b, which is necessary for the correct protein folding of proteins such as collagen type I^29^. Based on this evidence, we hypothesized that by silencing *TRAM2* in HeLa cells we may in turn block Ca^2+^ pumps of the ER (e.g. SERCA2b), and consequently alter the maturation of proteins that are essential for *S. aureus* during cell infection. In particular, we speculated with the possibility that TRAM2 depletion or SERCA inhibition may lead to an alteration in collagen type I folding and consequently its increased intracellular degradation, as previously reported^29^. This in turn could affect the adhesion of *S. aureus* to the host tissues through the collagen-binding adhesin Cna^30^, which may lead to a lower internalization of *S. aureus* into the host cell.

**Figure 5:**
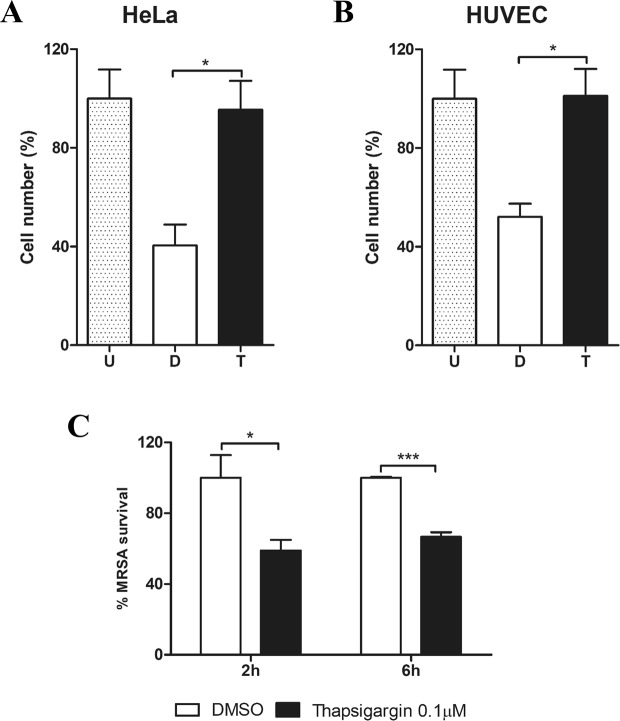
Thapsigargin treatment during *S. aureus* infection increases host cell viability whereas the intracellular bacterial load is reduced. HeLa cells and HUVEC were infected with *S. aureus* USA300-GFP (MOI 100) for 2 and 6 hours in the presence of DMSO (D) or 0.1 µM of Thapsigargin (T). Host cell viability in HeLa (**A**) and HUVEC (**B**) was quantified after 6 hours of infection by flow cytometry. Cell viability was normalized by the percentage of uninfected and untreated cells (U). (**C**) Intracellular MRSA survival was quantified by measuring the percentage of host cells with intracellular *S. aureus* USA300-GFP after 2 and 6 hours of infection. Data are expressed as means ± standard errors (SE) of at least two different experiments performed in duplicates and Student’s t-tests were performed to validate statistical significance across conditions. p-value ≤ 0.05 (*); ≤0.001 (***).

To test this hypothesis, we performed *S. aureus* infection assays in the presence of Thapsigargin, a selective SERCA inhibitor^31^, and we measured the host cell viability and intracellular MRSA survival. We employed a low concentration of this inhibitor (0.1 µM), because higher doses have been reported to be cytotoxic^32,33^. In the presence of Thapsigargin, host cell viability was enhanced at 6 hours post-infection both in human umbilical vein endothelial cells (HUVECs) and HeLa cells (Fig. 5). This is important, because host-cell interactions may profoundly differ depending on the cell line tested in the context of *S. aureus* cell infection^1^.

In concordance to the observed effect on host cell viability, intracellular MRSA survival was reduced by two-fold even at early time points of infection when infected HeLa cells where treated with Thapsigargin (Fig. 5C). On the other hand, we did not observe any significant differences in *S. aureus* growth curves *in vitro* in the presence of Thapsigargin, ruling out a direct inhibitory effect of the drug on *S. aureus* (Supplementary Fig. 6).

Our findings suggested that Thapsigargin halts *S. aureus* internalization in HeLa cells. However, this is not mediated by an altered production of collagen type 1, since COL1A2 (Collagen α2 Type I) levels were unaffected during *S. aureus* infection in wild type cells treated with Thapsigargin or in the TRAM2 knockdown, where TRAM2 protein levels are reduced by 50% when compared to a non-targeted control (Supplementary Fig. 7). Moreover, TRAM2 silencing did not have an effect during early time points of infection (i.e. 2 hours post-infection), pointing towards a role on intracellular proliferation rather than cell entry (Supplementary Fig. 8). Interestingly, autophagy is significantly reduced in response to TRAM2 depletion or Thapsigargin treatment in *S. aureus* infected cells (Supplementary Fig. 7e), suggesting a role of TRAM2 on the activation of the autophagic machinery.

### Characterization of the host gene *TRAM2* on *S. aureus* cell infection

To shed light on the role of TRAM2 on *S. aureus* intracellular survival, we have first evaluated the effect of *S. aureus* infection on the protein levels of TRAM2 in wild type HeLa cells at different time points and multiplicity of infections (MOI; Supplementary Fig. 9). As a control of bacterial load, we detected *S. aureus* Protein A by Western-blot, whose levels directly correlate with increasing times of infection and MOI (Supplementary Fig. 9a). Protein levels of TRAM2 were increased after 2 hours of *S. aureus* infection to become up to 40% higher in infected HeLa cells (Supplementary Fig. 9b). However, at 6 hours post-infection, we noticed a change in the migration pattern of TRAM2 upon denaturing protein electrophoresis that apparently generates a band of lower molecular weight, indicating that TRAM2 may have been subjected to post-translational modification such as proteolysis (Supplementary Fig. 9a). This change in the migration pattern was proportional to the MOI employed for the infection, suggesting that *S. aureus* may be interfering with the post-translational modification of TRAM2 to promote its own survival or replication. This strategy is commonly employed by many bacterial pathogens to control host proteins during intracellular infection^34^.

**Figure 6:**
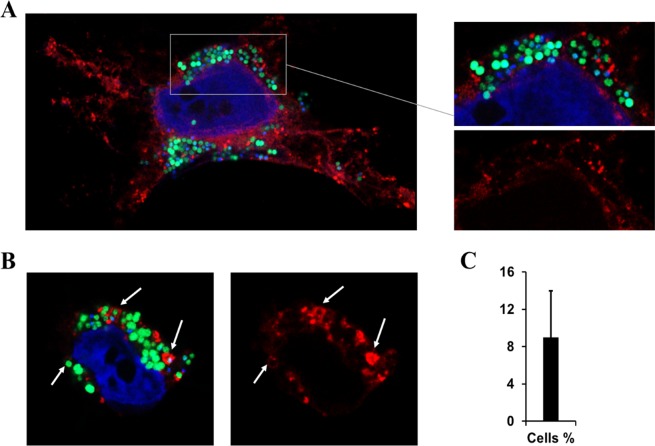
Co-localization of MRSA-GFP and TRAM2-mCherry by confocal microscopy. TRAM2-mCherry HeLa cells were infected with *S. aureus* USA300-GFP (MOI 100) and samples were collected after 6 hours of infection for confocal microscopy. DAPI staining was employed for nuclear staining. (**A**) Confocal picture showing a representative pattern of TRAM2-mCherry localization in *S. aureus* infected cells. (**B**) White arrows depict where co-localization between MRSA and TRAM2 was observed. (**C**) Percentage of infected cells showing co-localization of USA300-GFP and TRAM2-mCherry.

Given that TRAM2 is a membrane protein, we hypothesized that it may be involved in the maturation of the vacuole containing *S. aureus*. Therefore, HeLa cells stably expressing TRAM2-mCherry were infected with *S. aureus* USA300-GFP^35^. In most of the cases, we observed large sacs of *S. aureus* cells surrounded by a scattered pattern of TRAM2-mCherry (Fig. 6A), which was not considered a conclusive pattern of colocalization. However, some individual bacterial cells were enclosed by a membrane containing TRAM2-mCherry (Fig. 6B). Nevertheless, these clear co-localization patterns of *S. aureus*-TRAM2 were restricted to approximately 9% of infected cells (Fig. 6C). Taken together the low number of co-localization events, we can only conclude that either TRAM2 co-localization is highly dynamic or this protein may have other roles during cell infection rather than on the maturation of the vacuole containing intracellular *S. aureus*.

## Discussion

### A functional genomics approach identifies novel host-genes important for *S. aureus* during cell infection

The coupling of RNA interference (RNAi) with ultra-fast sequencing provides an exceptional opportunity to simultaneously screen the implication of thousands of host-genes in different areas of research. In particular, RNAi approaches have been employed before to screen novel host-genes implicated in intracellular bacterial^36^, parasitical^37^ and viral^38^ infections.

The main limitation of this high-throughput approach is the possibility of encountering phenotypes caused by off-target effects. To minimize this, transductions should be performed with a low multiplicity of infection^14^, and different shRNA constructs targeting the same gene should be included in the pooled libraries^16^. Accordingly, we performed lentiviral transductions using a MOI of 1, and most genes included in the commercial shRNA library were targeted by at least 5 shRNA constructs.

By employing ultra-fast sequencing, we were able to rapidly estimate the occurrence of shRNAs in *S. aureus*-infected cells in relation to the uninfected control. Over-represented shRNA constructs may be silencing human genes that are important for the intracellular bacterial survival and thus, any of these genes could have an essential role on *S. aureus* cell infection. We found approximately 10,000 human genes that were targeted by shRNAs over-represented in HeLa cells at 6 hours post-infection.

Further validation of our preliminary screening results was necessary and thus, the number of potential candidates was filtered. A combination of high consistency of the silencing effect, magnitude of the change and biological relevance was employed to select genes for further downstream analysis. As a result, 29 human genes were selected for further validation by producing individual knockdowns in HeLa cells and testing host cell viability after intracellular MRSA infection. Among these genes, we included a varied range of cellular processes, such as autophagy, apoptosis, membrane trafficking and cytoskeleton rearrangement, amino-acids metabolism and host-immune responses. Many of these host cell pathways have previously been described to play a role in intracellular infection and therefore, we evaluated how host cell viability was affected during *S. aureus* infection after individually silencing these host-genes.

Of particular significance is ATG10, which silencing significantly restored cell viability after infection with two different MRSA strains (Fig. 3). This is in agreement with findings from our and other groups, suggesting that intracellular *S. aureus* infection in non-phagocytic cells is halted with autophagy inhibitors such as dorsomorphin^17^, while 3-Methyladenine treatment protects mice from MRSA pneumonia^39^.

To further identify other host-targeted therapeutics against intracellular MRSA, we interrogated our shRNA screening results to identify four genes, i.e. *CD83, FAM63B, MYL2* and *TRAM2*, whose silencing in HeLa cells resulted in a significant reduction of intracellular *S. aureus* survival while host cell viability was enhanced after cell infection.

The protein encoded by *CD83* gene is a host membrane protein that is part of the immunoglobulin family and it is mainly expressed in mature dendritic cells (DCs). The main function of these antigen-presenting cells is the activation of host immune responses against invading pathogens^40^. Eukaryotic cells have evolved complex immune mechanisms to combat invading pathogens. Consequently, and in order to successfully establish infections, intracellular microorganisms have also developed sophisticated mechanisms to subvert and overcome host immune responses^41^. Specifically, it has been shown that interleukin 10 (IL-10) – a cytokine produced by macrophages, T-cells, B-cells, mast cells and keratinocytes, among others – can be an effective immunosuppressive factor and therefore, the exploitation of this cytokine is a common mechanism to evade host immune responses by several pathogens. Some viruses induce the production of host IL-10, while others produce their own IL-10-homologs. Bacterial intracellular pathogens, such as *Mycobacterium tuberculosis* and *Listeria monocytogenes*, are able to replicate within macrophages by inducing IL-10^42^. However, further investigation is needed to explain the molecular mechanism behind *CD83*-*S. aureus* interaction in cells that are not part of the immune system.

Although its function is unknown, FAM63B has been recently identified as an interaction partner of kinesin light chain-1 (KLC1), which is involved in the intracellular trafficking of vaccinia virus^43^. Kinesins, dyneins and myosins comprise three classes of molecular motors that are part of the host cell cytoskeleton and are involved in many biological processes related to cell movement. Intracellular bacterial pathogens and viruses commonly use this host machinery to reach their intracellular niche, as well as to control the membrane remodelling of their containing vacuoles^44^. In particular, Kinesin-1 has been implicated in the intracellular dissemination of adenovirus and Herpes virus^43,45,46^. Moreover, *Salmonella* effector protein SifA targets the host protein SKIP, which down-regulates the recruitment of Kinesin-1 to the *Salmonella*-containing vacuole and thus, controls its dynamics^47^. Therefore, FAM63B may play a role in the intracellular trafficking of *S. aureus* by targeting kinesin proteins.

Myosins comprise a superfamily of motor proteins found in actin filaments and have an essential role in the organization of actin cytoskeleton. Polymerization and depolymerization of actin filaments promote changes in cells shape and, along with myosin proteins, control the intracellular organization^25^. The host cell cytoskeleton is commonly hijacked by intracellular pathogens to support their own intracellular invasion, survival and replication and actin has been specifically described as a common target of many bacterial pathogens^48^. As above mentioned, bacterial pathogens exploit host-actin for different purposes such as intracellular invasion, actin-based motility through cell cytoplasm and pathogen dissemination^49^. Likewise, the use of microtubules, motor proteins and intermediate filaments in intracellular invasion and dissemination has been highlighted^49^. For instance, the actin-base motor protein myosin 2 has been associated with the integrity and location of *Salmonella*-containing vacuoles^50^. In addition, myosin 2 has been associated with the promotion of vesicle fission from the Golgi complex^51^. Furthermore, there is a proportion of cytosolic *Shigella* that become covered with septin filaments in a myosin 2-dependent manner^52^. Moreover, the release of *Chlamydia-*intrusions into the extracellular space is mediated by actin, N-WASP, myosin 2 and GTPase Rho proteins^53^. Collectively, these studies suggest that the intracellular fate of many intracellular pathogens is determined by an organized collaboration between actin, microtubules, intermediate filaments and motor proteins. Therefore, the gene coding for the myosin light chains 2 (MYL2) could be involved in the intracellular dissemination of *S. aureus* by controlling host actin cytoskeleton. However, a treatment with the myosin II inhibitor Blebbistatin did not restore the host cell viability during *S. aureus* infection (Supplementary Fig. 5), suggesting a more complex scenario.

### TRAM2 plays an important role during intracellular MRSA survival

TRAM2 (Translocating chain-associated membrane protein 2) was initially identified as being responsible for the translocation of proteins across the endoplasmic reticulum membrane^54^. Furthermore, TRAM2 interacts with the endoplasmic reticulum Ca^2+^ ATPase transporter SERCA2b, being involved in collagen type I protein folding^29^.

Different intracellular pathogens rely on the endoplasmic reticulum and its components to ensure intracellular survival and proliferation^55^. For instance, *Legionella pneumophila*, an intravacuolar pathogen, hijacks host membrane components to contribute to the formation of *Legionella*-containing vacuoles that are mainly derived from endoplasmic reticulum material^56,57^. Similarly, *Brucella abortus*^58,59^ and *Chamydia trachomatis*^60,61^ also exploit host-endoplasmic reticulum to promote their intracellular survival and replication within mammalian cells. In addition, SERCA2b has been closely associated with Chlamydial infections in HeLa cells^62^. In concordance with these observations, co-localization assays showed that several chlamydial antigens, such as major outer membrane protein (MOMP), inclusion membrane protein and lipopolysaccharides (LPS), were specifically associated with the ER and ER-markers^63^.

Because of the interaction of TRAM2 and SERCA transporters, we investigated the intracellular *S. aureus* survival under treatment with Thapsigargin – a promising anticancer drug that is a specific host-SERCA inhibitor^31,64^. High doses of Thapsigargin lead to host cell death due to the ER stress caused by this inhibitor^33^. In contrast, treatment of infected HeLa cells with low doses of Thapsigargin resulted in a significantly attenuated bacterial load at both time points of infection (at 2 and 6 hours post infection, Fig. 5), whereas this reduction was only observed after 6 hours of infection in TRAM2 knockdown cells (Supplementary Fig. 8). Therefore, Thapsigargin may have an effect on cell entry, whereas TRAM2 depletion is not altering cell invasion but it can halt *S. aureus* intracellular replication. Nonetheless, our findings suggest that the anti-cancer drug Thapsigargin may be repurposed to block *S. aureus* cell infection when combined with conventional antibiotics. On the other hand, neither TRAM2 depletion nor Thapsigargin treatment had any effects on type I collagen production during host cell infection, pointing towards an alternative role of TRAM2 on *S. aureus* intracellular survival in this context. In concordance with this hypothesis, we have not observed any significant changes in the occurrence of shRNAs in *S. aureus*-infected cells that are silencing essential components of the translocon such as SEC61A1 or SEC61G (Supplementary Table 1), suggesting that the role of TRAM2 on the intracellular survival of *S. aureus* is not related to protein translocation across the endoplasmic reticulum membrane. Interestingly, we have observed autophagy inhibition during *S. aureus* infection in the context of TRAM2 depletion or Thapsigargin treatment, but this effect may be indirect due to a lower bacterial load. Undoubtedly, more research is needed to fully understand the role of TRAM2 during *S. aureus* infection.

## Conclusions

By using a functional genomics approach, we screened the implication of 16,000 host-genes on intracellular *S. aureus* infection to identify many potential host targets that could be hijacked by *S. aureus* during intracellular infection. Here, we studied the human gene *TRAM2*, which plays an important role during *S. aureus* infection. The data presented in this study serve as proof-of-principle of a functional genomics approach to unravel novel molecular mechanisms hijacked by *S. aureus* during intracellular proliferation in human cells.

## Materials and Methods

### Bacterial strains, cell lines and culture conditions

*S. aureus* NCTC 13626^65^, USA300 LAC^66^ and USA300-GFP^35^ strains were cultured and pre-inocula for infection assays were prepared as previously described^17^. Briefly, bacterial culture was grown from an overnight culture until an optical density (OD600 nm) of 1 was reached, which corresponds to the mid-exponential phase (Supplementary Fig. 10). Cultures were centrifuged, and pellets were washed before being resuspended in PBS supplemented with 20% glycerol to aliquot and store at −80 °C until needed.

HeLa cells (ECACC 93021013) and their derivatives were incubated at 37 °C and 5% of CO_2_ in Dulbecco’s Modified Eagle’s medium (DMEM, Gibco) containing pyruvate, glucose and glutamine and supplemented with 10% heat-inactivated foetal bovine serum (FBS, Gibco) and 5% of penicillin and streptomycin solution (Gibco), unless otherwise specified. Human umbilical vein endothelial cells (HUVECs; Sigma) were grown in endothelial cell growth medium (Sigma).

### shRNA screening

HeLa cells were transduced with the Mission^®^ LentiPlex Human shRNA Library (Sigma-Aldrich). This library is comprised by 10 sub-pools of lentiviral particles carrying around 75,000 shRNA constructs that target 16,000 human genes. HeLa cells were seeded in ten different 100 mm plates (Sarstedt) in complete DMEM medium at a cell density of 2 × 10^6^ cells per dish and placed in the incubator overnight (37C, 5% CO_2_). The following day, the ten-different lentiviral subpools were thawed on ice and added to the HeLa cells at a MOI of 1 in the presence of hexadimethrine bromide (8 µg/ml; Sigma-Aldrich). After an overnight incubation, the virus-containing media were replaced with fresh DMEM without hexadimethrine bromide. The next day, HeLa cells were exposed to puromycin selection (1 µg/ml; Sigma-Aldrich) and medium was replaced with fresh puromycin-containing media every two days until only positive transduced-cells remained.

HeLa cell lines stably expressing the shRNA library were infected with *S. aureus* NCTC 13626 at a MOI of 100. After 6 hours of infection, total DNA was extracted from uninfected and *S. aureus*-infected cells by using GenElute Mammalian Genomic DNA Miniprep Kit (Sigma-Aldrich), following manufacturer’s instructions. Genomic DNA samples were submitted to the Sigma Deconvolution platform, and the abundance of each shRNA clone in samples was tested by amplifying and next-generation sequencing (at × 1,000) shRNA regions and barcoding samples; short reads were aligned to the reference. Data were obtained as the number of shRNA sequences per clone per sample, as previously described^67^. Screening results are shown in Supplementary Table 1.

### Production of individual knockdowns in HeLa cells

To produce individual knockdowns, HeLa cells were transduced with 29 individual shRNA lentiviral constructs (Supplementary Table 4, Sigma-Aldrich). HeLa cells were seeded in 24 wells-plates at a cell density of 8 × 10^4^ cells per well and incubated overnight (37 °C, 5% CO_2_). Transduction of mammalian cells was performed as described above, employing a MOI of 1.

HeLa cells expressing TRAM2-mCherry were created by transduction with P12-MMP-TRAM2-mCherry, as previously described^68^. To create this vector, mCherry-LC3 was replaced with TRAM2-mCherry in P12-MMP-mCherry-LC3^68^. TRAM2 was amplified from the cDNA Clone IRATp970H1249D (SourceBioscience) with primers TAGCTAAAGCTTGCCACCATGGCTTTCCGCAGGAGG and GCCCTTGCTCACCATGGGAGACTTGAGTTT, whereas mCherry was amplified from P12-MMP-mCherry-LC3 with AAACTCAAGTCTCCCATGGTGAGCAAGGGC and TAGCTAGCGGCCGCTTTACTTGTACAGCTCGTCCAT, and subjected to fusion PCR. The DNA amplicon was verified by sequencing, digested with HindIII/NotI, and cloned into P12-MMP to create P12-MMP-TRAM2-mCherry.

### Intracellular infection assays

Intracellular infection assays were performed as described before^17^. For host cell viability assays, cells were double stained with annexin V-FITC and propidium iodide according to manufacturer’s recommendations (Becton Dickinson, BD), and host cell viability was measured by flow cytometry (BD Accuri^TM^ C6 Plus) as previously described^17^. To estimate the intracellular bacterial load at specific time points after infection, cells were lysed using 0.1% Triton X-100 diluted in PBS (5 min, RT) and serial dilutions were plated in Nutrient Agar plates for colony forming unit (CFU) counting. For assays involving Thapsigargin treatment, a flow cytometry-based protocol developed for drug screening was applied as previously described^18^.

### Western-blot

Western-blots were performed as described before^17^. Primary antibodies were purchased from Santa Cruz Biotechnologies (anti-GAPDH [reference no. sc-47724]; anti-COL1A2 [sc-166865]), Fisher (anti-LC3 [13278218]), and Novus Biologicals (anti-TRAM2 [NBP1-83052]). Secondary antibodies were purchased from Li-Cor (IRDye 680LT goat anti-mouse [926–68070] and IRDye 800LT goat anti-rabbit [926–32211]). Full-length blots are shown in Supplementary Fig. 11.

### Confocal microscopy

HeLa cells expressing TRAM2-mCherry were seeded in coverslips at a cell density of 7 × 10^4^ cells per well in 24 well plates and *S. aureus* USA300-GFP^35^ was used to infect the cells at a MOI of 100 as previously described^18^. After 6 hours of infection, cells were fixed with 0.5 ml of freshly prepared 4% of paraformaldehyde (PFA; Fisher Scientific) for 15 minutes. Coverslips were then washed twice with PBS and mounted on microscope slides (Thermo Fisher Scientific) using ProLong Gold Antifade mountant with DAPI (Thermo Fisher Scientific) for nuclear staining. Slides were observed on a confocal microscope ZEISS LSM 800 with Airyscan by using Zen Blue Software (Zeiss).

### Statistical analyses

Statistical tests and graphs plotting were conducted using GraphPad Prism software and significant differences across treatments were assessed by running Student’s t-test and *post hoc* Tukey’s multiple comparison tests when required.

## Supplementary information

Supplementary Figures and Tables

Dataset 1

Dataset 2
