# Supplementary material for: Identification of novel targets for host-directed therapeutics against intracellular Staphylococcus aureus
Source: Sci Rep. 2019 Oct 28;9:15435. doi: 10.1038/s41598-019-51894-3 (PMC6817851; doi:10.1038/s41598-019-51894-3)
Supplement: Supplementary file 1 — Supplementary Figures and Tables [file 41598_2019_51894_MOESM1_ESM.pdf]

Identification of novel targets for host-directed therapeutics against intracellular *Staphylococcus aureus*

Natalia Bravo-Santano[a], Pablo Capilla-Lasheras[b], Luis M. Mateos[c], Yolanda Calle[a], Volker Behrends[a#], Michal Letek[a#]

[a]Health Sciences Research Centre, University of Roehampton, London, UK.

[b]Centre for Ecology and Conservation, University of Exeter, Penryn Campus, Cornwall, UK.

[c]Department of Molecular Biology, Area of Microbiology, University of León, León, Spain

#Address correspondence to Volker Behrends, Volker.Behrends@roehampton.ac.uk and Michal Letek, Michal.Letek@roehampton.ac.uk

SUPPLEMENTARY FIGURES

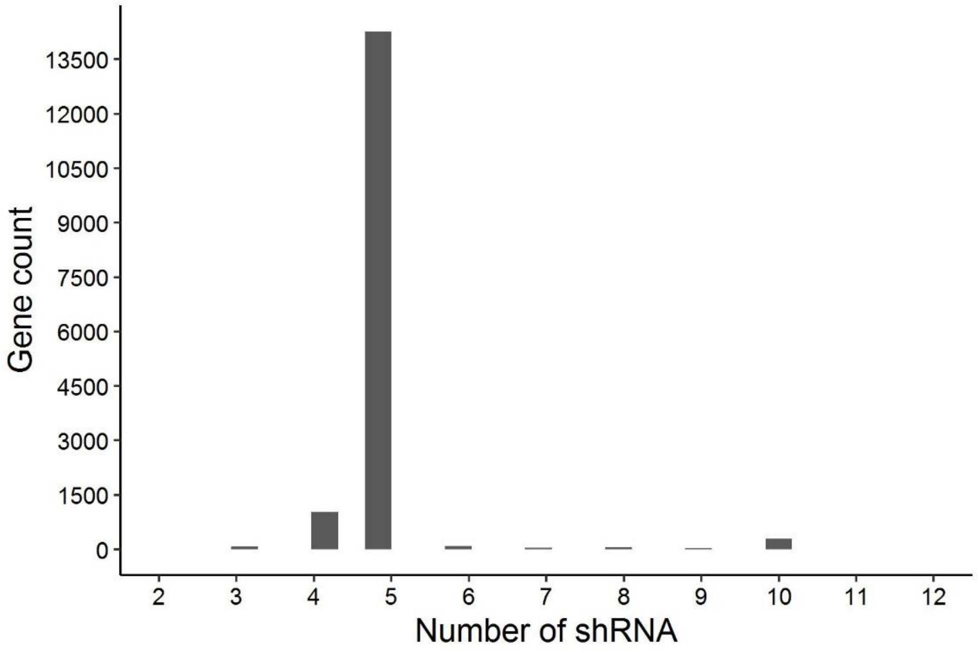

Supplementary Figure 1. The Mission® LentiPlex® Human Pooled shRNA Library comprises over 75,000 shRNA constructs that were divided in 10 different sub-pools. The figure shows the distribution of shRNA constructs included in the library. Over 13,500 genes were targeted by at least 5 different shRNA constructs**.**

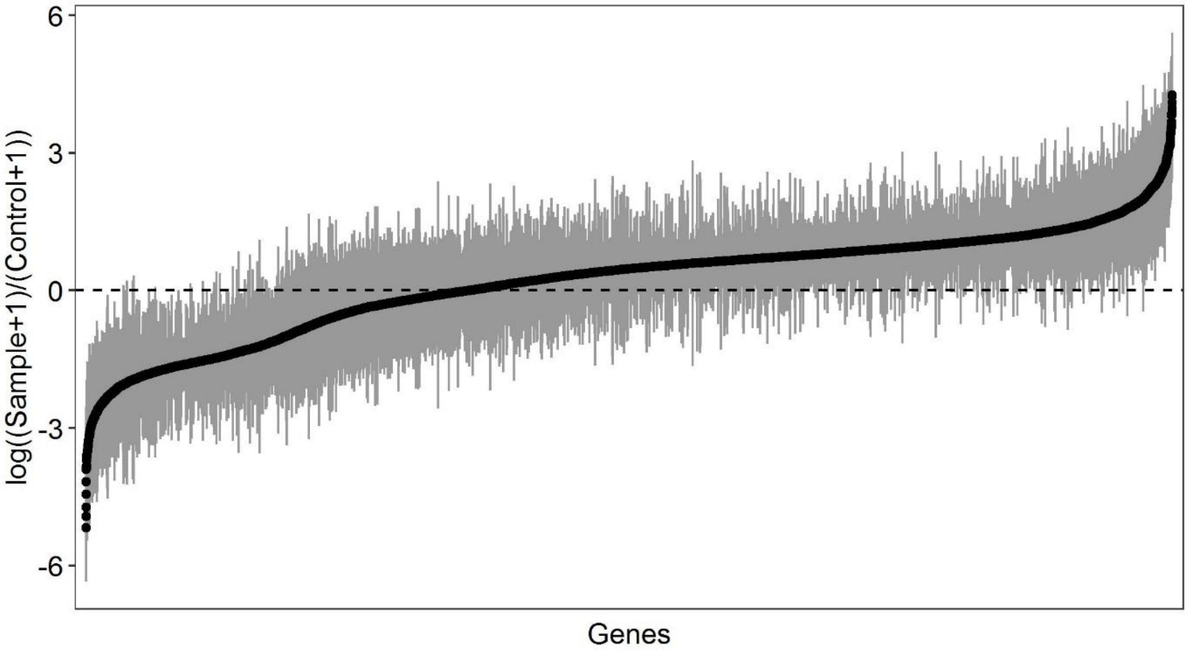

Supplementary Figure 2. shRNA screening in HeLa cells after *S. aureus* infection. The relative quantity of each shRNA found in MRSA-infected cells was divided by the relative quantity found in uninfected cells and values were Log-transformed (Log+1). Mean and standard error (SE) were calculated for those shRNAs that were targeting the same gene and plotted into the above graph. Means and standard errors are represented as black dots and grey lines, respectively. Black dots become as a continuous black line due to the high number of genes.

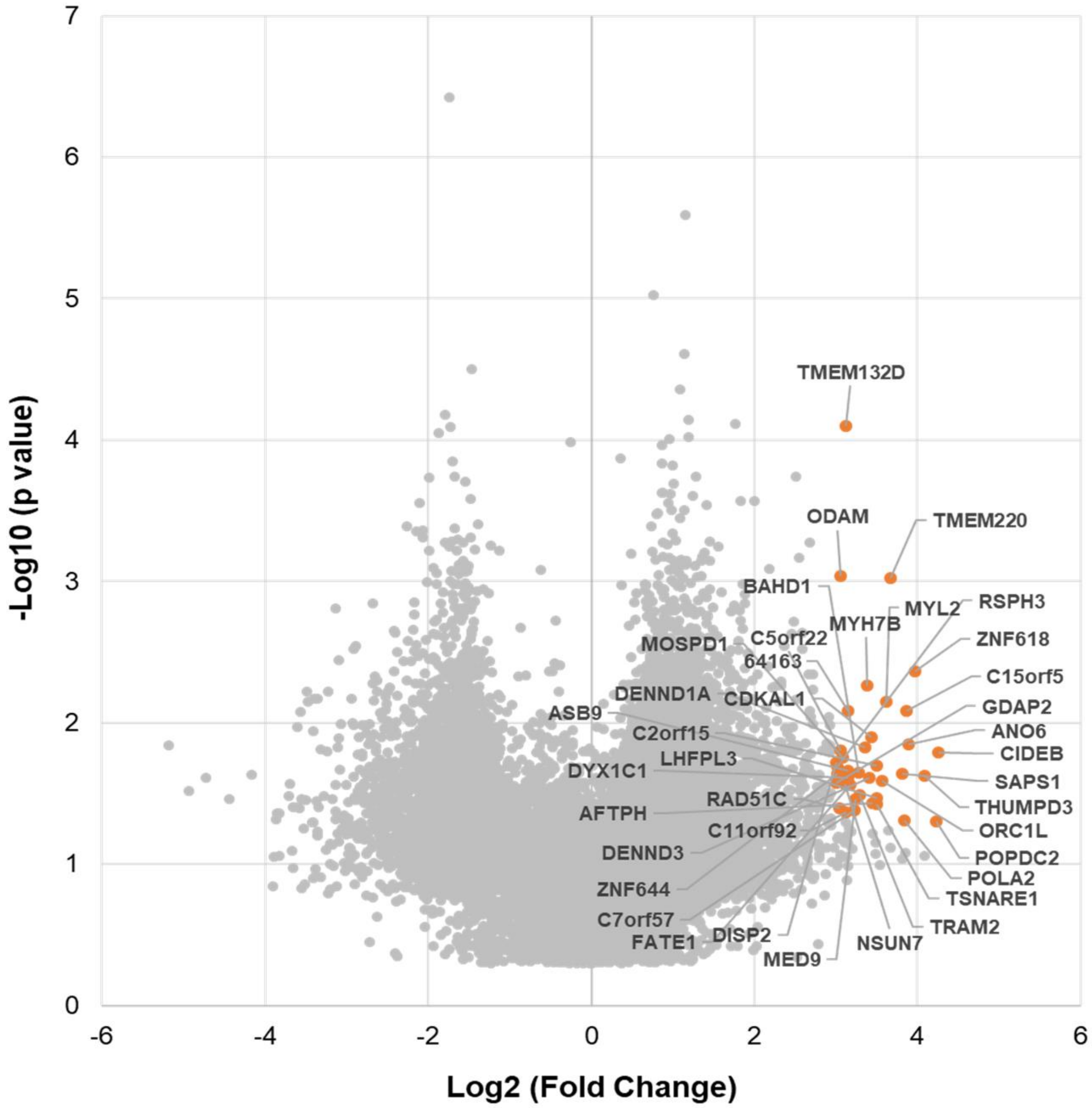

Supplementary Figure 3. Volcano-plot of the shRNA screen results. Screening results of 16,000 genes are ranked by fold-change and statistical significance. Orange dots comprise genes with Log_2 fold change of the mean ratio above 3 and $-Log_{10}$ p-value higher than 1.3.

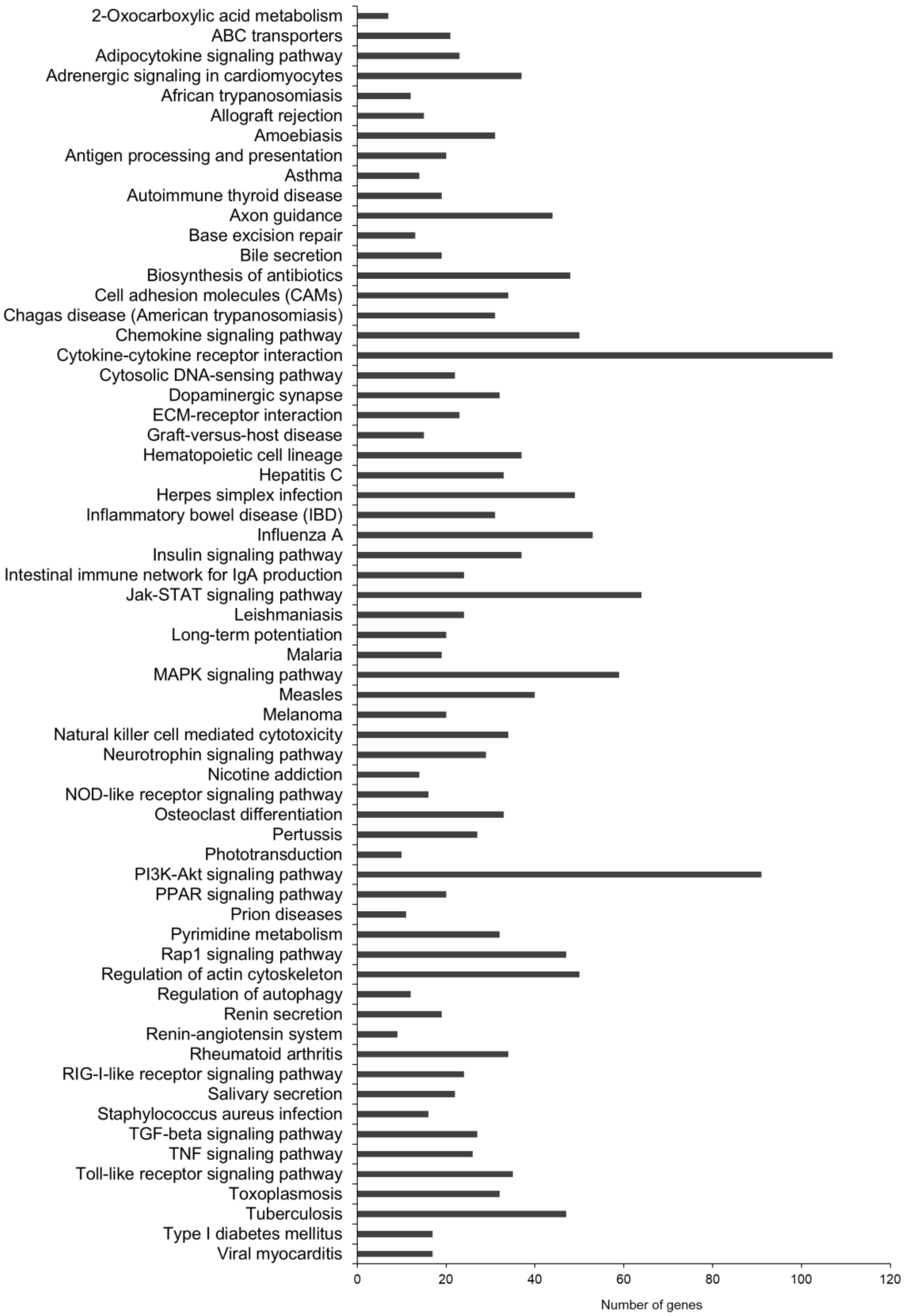

Supplementary Figure 4. Functional classification of genes identified during the shRNA screen. KEGG pathways were analysed employing DAVID Bioinformatics Resources 6.8.

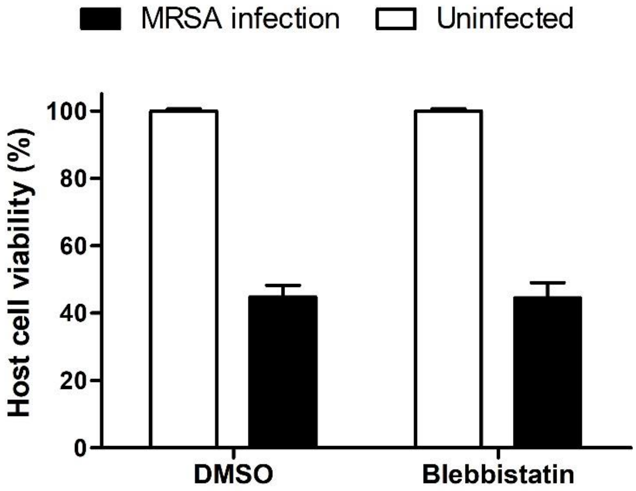

Supplementary Figure 5. Host cell viability in HeLa cells under blebbistatin treatment. Cell viability was quantified after 6 hours of infection by flow cytometry, using a double staining of Annexin V-FITC and propidium iodide. Uninfected HeLa cells were employed as negative control and cell viability was normalized in relation to control´s viability. Data are expressed by means ± standard error (SE) of three experiments performed in duplicates. No significant differences were found among treatments.

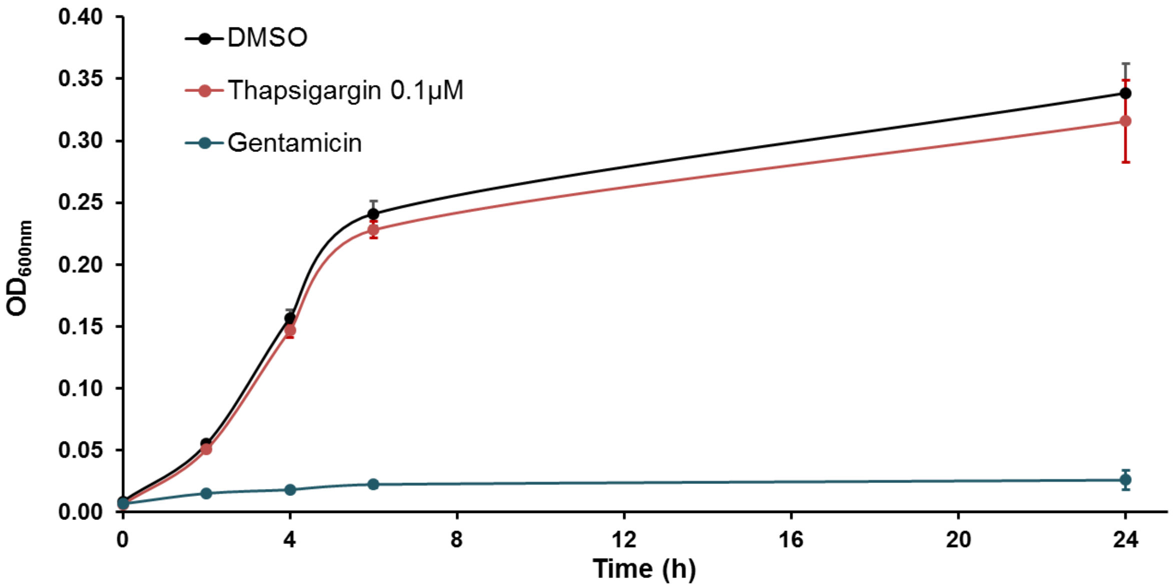

Supplementary Figure 6. Thapsigargin does not directly affect in vitro *S. aureus* growth. *S. aureus* USA300 was grown in the presence of DMSO, Thapsigargin (0.1 μM) or gentamicin. Absorbance (OD_{600nm}) was measured at 2, 4, 6 and 24 hours to produce bacterial growth curves. Data are expressed as means ± standard errors of three different experiments performed in triplicates.

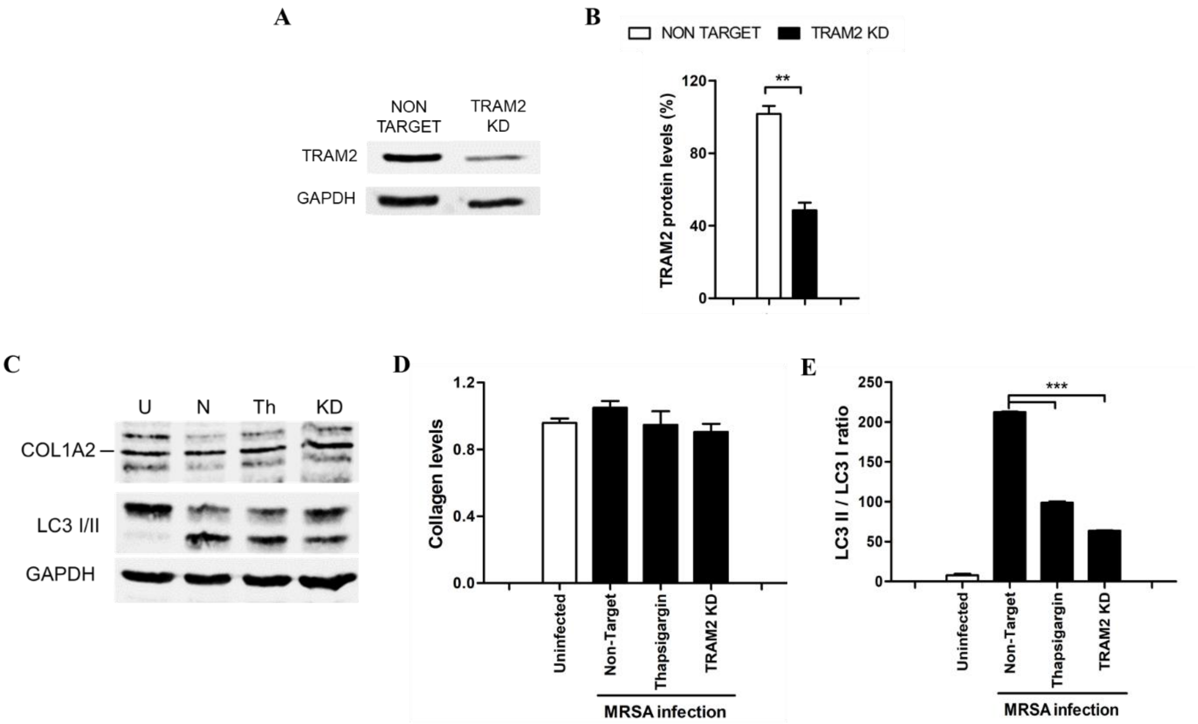

Supplementary Figure 7. Validation of *TRAM2* Knockdown and quantification of collagen α2 Type I (COL1A2) and LC3 I/II protein levels after MRSA infection. (a) Cell extracts were analysed by Western-blot against TRAM2, and GAPDH was used as loading control. (b) Quantification of TRAM2 protein levels observed by Western-blot. (c, d and e) Non-Target untreated HeLa cells (N), Non-Target cells under Thapsigargin treatment (Th) and TRAM2 KD cells (KD) were infected with MRSA USA300 and cell extracts were collected after 6 hours of infection. Uninfected Non-Target HeLa cells (U) were used as negative control. (c) Cell extracts were analysed by Western-blot against collagen (COL1A2) or LC3, and GAPDH was used as loading control. (d) Quantification of collagen protein levels observed by Western-blot. (e) Quantification of the conversion of LC3-I to LC3-II. Data are normalized to GAPDH levels and expressed as means ± standard errors (SE) of three biological replicates. One-way ANOVA and multiple comparison Tukey´s tests were performed to assess statistically significant differences between each knockdown and the non-target control. p-value ≤0.001 (***). Full-length blots are shown in Supplementary Figure 11.

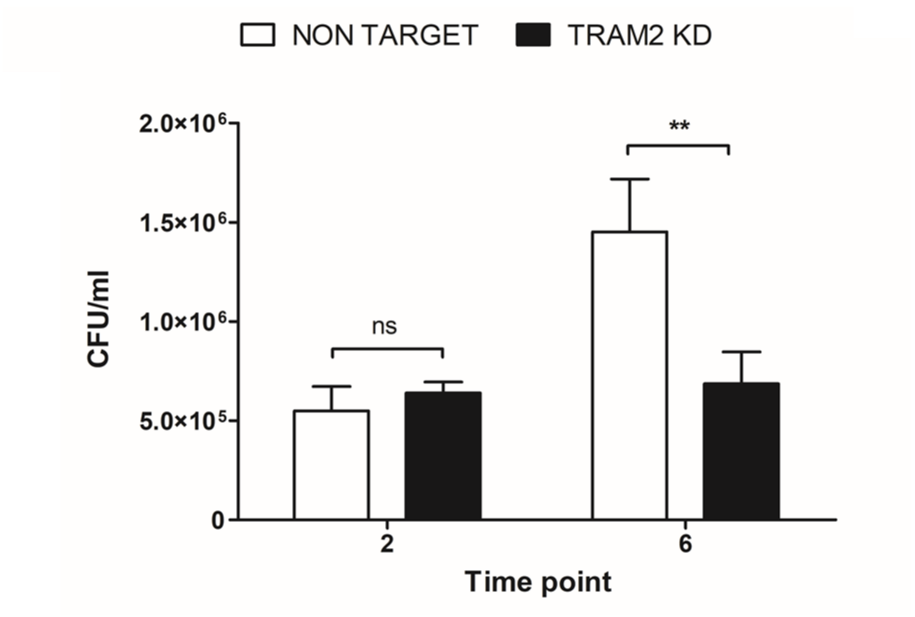

Supplementary Figure 8. Intracellular MRSA infection is significantly hampered after 6 hours of infection. Non-target and TRAM2 KD HeLa cells were infected with MRSA (MOI 100) and colony forming units (CFU) were counted after 2 and 6 hours of infection. Data are expressed as means ± standard errors (SE) of three biological replicates performed in duplicates. Student´s t-tests were performed to validate statistical significance across conditions; p-value ≤0.01 (**).

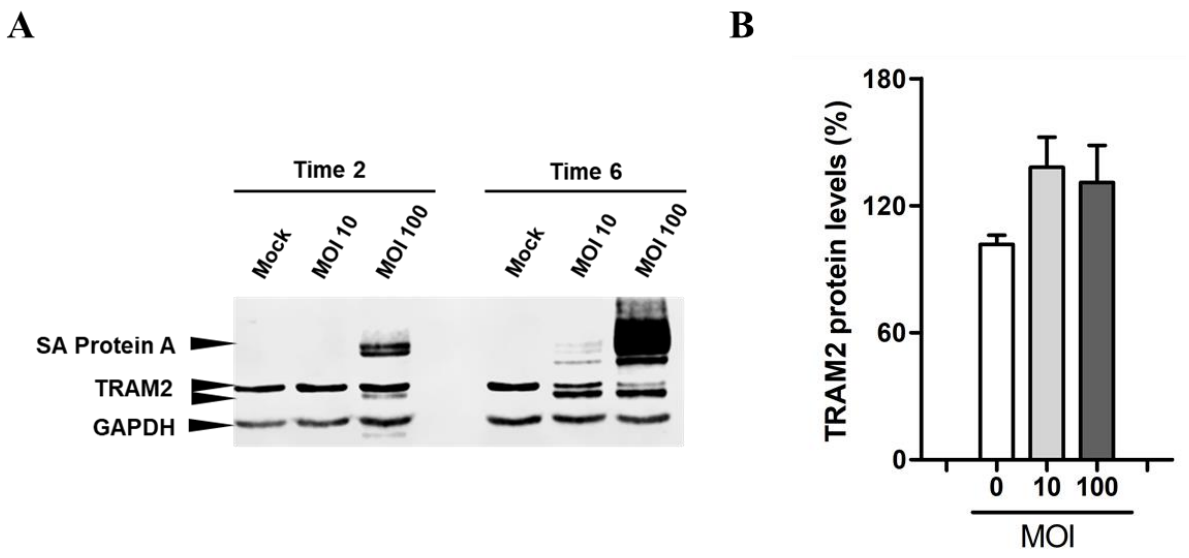

Supplementary Figure 9. TRAM2 protein levels in HeLa cells during *S. aureus* cell infection. HeLa cells were infected with MRSA USA300 (MOI 10 and 100) and cell lysates were collected after 2 and 6 hours post-infection. (a) Cell extracts were analysed by Western-blot against TRAM2 and GAPDH antibody was employed as loading control. (b) Quantification of TRAM2 protein levels observed by Western-blot at time point 2. Data are normalized to GAPDH levels and are expressed as means ± standard errors (SE) of three biological replicates. Student´s t-tests were performed to validate statistical significance across conditions. p-value ≤0.01 (**). Uninfected cells (Mock) were used as negative control. Full-length blots are shown in Supplementary Figure 11.

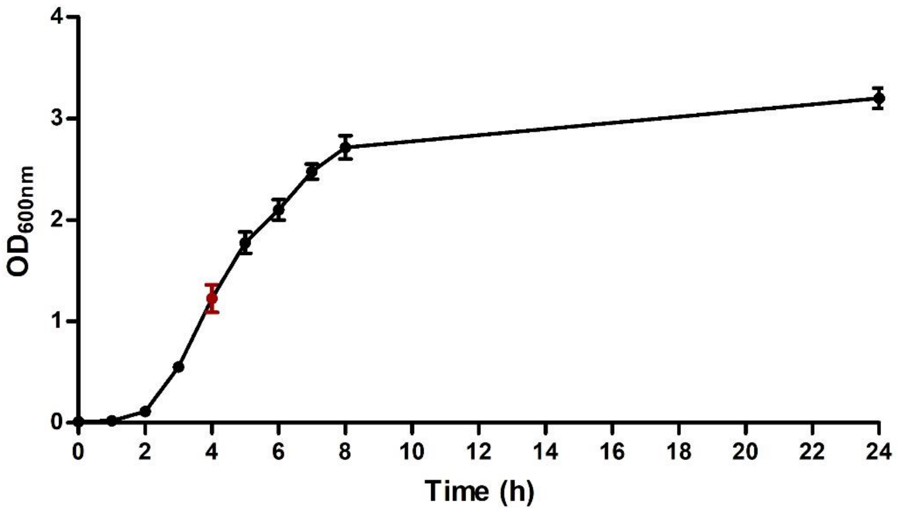

Supplementary Figure 10. In vitro MRSA USA300 growth in Nutrient Broth. *S. aureus* USA300 was grown in NB and absorbance (OD_{600nm}) was measured at 1, 2, 3, 4, 5, 6, 7, 8 and 24 hours to produce bacterial growth curves. Data are expressed as means ± standard errors of three different experiments performed in triplicates. OD~1 – which we routinely employed for intracellular infection assays – was reached after 4 hours and it is highlighted in red.

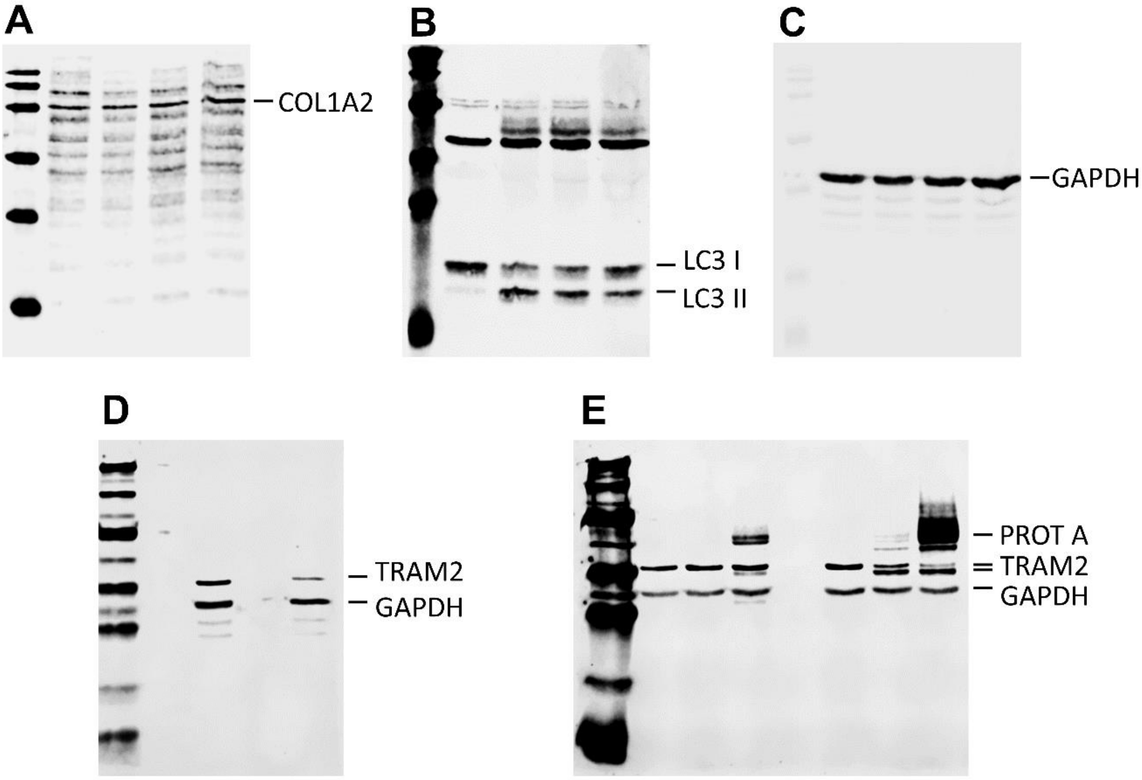

80 **Supplementary Figure 11.** Full length blots of Supplementary Figures 7 (A-C) and 9 (D-E).

SUPPLEMENTARY TABLES

Caption for Supplementary Table 1.xlsx

Supplementary Table 1. Analysis of the shRNA screening results. Each gene included in the screen is identified by its NCBI ID and a consolidated name. The analysis of the results includes the number of shRNAs targeting a given gene, the number of shRNA producing a positive or negative sample-to-control ratio (positive and negative hits, respectively), and the consistency of the silencing effect (percentage of shRNAs that produced ratios in the same direction, either positive or negative). The relative quantity of each shRNA found in MRSA-infected cells was divided by the relative quantity found in uninfected cells and values were Log-transformed (Log+1); the log_2 fold-change, standard deviation, standard error and $-Log_{10}$ of p-value were calculated for each gene to determine the biological significance and the magnitude of change.

Caption for Supplementary Table 2.xlsx

Supplementary Table 2. Functional classification of the genes targeted by shRNAs over-represented after *S. aureus* infection. Genes were classified by KEGG pathways through DAVID Bioinformatics Resources 6.8. KEGG pathways were identified by their name and entry code. The number of genes per pathway and the names of the genes classified in each pathway are listed.

NCBI GeneID	Gene	Number shRNA	Positive hits	Negative hits	Consistency (%)	Mean ratio	SE ratio
83734	*ATG10*	5	4	1	80	1.002	1.748
57099	*AVEN*	5	3	0	60	1.083	0.649
10295	*BCKDK*	9	8	1	89	1.365	0.405
79780	*CCDC82*	5	4	0	80	3.030	1.248
922	*CD5L*	5	4	1	80	2.379	1.126
9308	*CD83*	5	4	1	80	1.250	1.404
54901	*CDKAL1*	5	5	0	100	3.438	1.149
1212	*CLTB*	5	3	1	60	1.581	0.823
1438	*CSF2RA*	5	4	0	80	2.259	0.921
54629	*FAM63B*	5	5	0	100	2.596	1.212
439996	*IFIT1B*	5	4	1	80	2.441	1.207
3775	*KCNK1*	5	5	0	100	2.869	0.930
1955	*MEGF9*	5	4	0	80	2.638	0.806
4633	*MYL2*	5	5	0	100	3.624	1.045
10398	*MYL9*	5	5	0	100	2.938	0.733
4753	*NELL2*	5	4	0	80	2.095	0.804
79730	*NSUN7*	5	5	0	100	3.111	0.883
4998	*ORC1L*	5	5	0	100	3.571	0.646
23649	*POLA2*	5	5	0	100	3.838	1.002
84440	*RAB11FIP4*	5	4	1	80	1.161	0.419
25782	*RAB3GAP2*	5	3	2	60	1.028	0.632
347517	*RAB41*	5	5	0	100	2.692	1.102
285641	*SLC36A3*	5	5	0	100	2.915	0.780
8501	*SLC43A1*	5	5	0	100	3.123	0.878
7097	*TLR2*	5	5	0	100	2.179	1.141
8794	*TNFRSF10C*	5	5	0	100	2.104	0.503
9697	*TRAM2*	5	5	0	100	3.292	0.601
127733	*UBXN10*	5	4	1	80	1.813	0.517
114991	*ZNF618*	5	5	0	100	3.970	0.732

Supplementary Table 3. Twenty-nine genes were selected from the shRNA screening for further validation. Each gene is identified with its name along with the NCBI identification number. Positive hits, refers to the shRNAs constructs whose ratio was a positive number and thus, the representation of that shRNA construct was higher in the MRSA-infected sample than the uninfected control. By contrast, negative hits include shRNAs whose quantification in the MRSA-infected sample was lower than the uninfected sample. Consistency is the percentage of shRNAs that produce a similar silencing effect.

Means and standard errors (SE) were calculated for the different shRNAs constructs that target the same gene.

Clone ID	Symbol	Gene description
TRCN0000127872	*ATG10*	ATG10 autophagy-related 10 homolog
TRCN0000122869	*AVEN*	Apoptosis; caspase activation inhibitor
TRCN0000199388	*BCKDK*	Branched chain ketoacid dehydrogenase kinase
TRCN0000130856	*CCDC82*	Coiled-coil domain containing 82
TRCN0000057589	*CD5L*	CD5 molecule-like
TRCN0000056919	*CD83*	CD83 molecule
TRCN0000056647	*CDKAL1*	CDK5 regulatory subunit associated protein 1
TRCN0000055994	*CLTB*	Clathrin; light polypeptide (Lcb)
TRCN0000058248	*CSF2RA*	Colony stimulating factor 2 receptor alpha; low affinity
TRCN0000142964	*FAM63B*	Family with sequence similarity 63, member B
TRCN0000129900	*IFIT1B*	Interferon-induced protein with tetratricopeptides repeats 1-like
TRCN0000044726	*KCNK1*	Potassium channel; subfamily K; member 1
TRCN0000055585	*MEGF9*	Multiple epidermal growth factor-like; domain 9
TRCN0000053959	*MYL2*	Myosin; Light polypeptide 2; regulatory; cardiac; slow
TRCN0000053505	*MYL9*	Myosin; light polypeptide 9; regulatory
TRCN0000054308	*NELL2*	NEL-like 2
TRCN0000138344	*NSUN7*	NOL1/NOP2/Sun domain family; member 7
TRCN0000144974	*ORC1L*	Origin recognition complex; subunit 1-like
TRCN0000053075	*POLA2*	Polymerase (DNA directed); alpha 2
TRCN0000056506	*RAB11FIP4*	RAB11 family interacting protein 4 (Class II)
TRCN0000047220	*RAB3GAP2*	RAB3 GTPase activating protein subunit 2 (non-catalytic)
TRCN0000141304	*RAB41*	RAB41; member RAS; oncogene family
TRCN0000044061	*SLC36A3*	Solute carrier family 36 (proton/amino acid symporter), member 3
TRCN0000044314	*SLC43A1*	Solute carrier family 43; member 1
TRCN0000057018	*TLR2*	Toll-like receptor 2
TRCN0000059251	*TNFRSF10C*	Tumor necrosis factor receptor superfamily; member 10C
TRCN0000141247	*TRAM2*	Translocation associated membrane protein 2
TRCN0000011171	*UBXN10*	UBX domain containing 3
TRCN0000137602	*ZNF618*	Zinc finger protein 618

Supplementary Table 4. List of lentiviral particles employed to produce individual knockdowns in HeLa cells.
